# Students’ and supervisors’ knowledge and attitudes regarding plagiarism and referencing

**DOI:** 10.1186/s41073-018-0054-2

**Published:** 2018-10-23

**Authors:** Johanna F Lindahl, Delia Grace

**Affiliations:** 1 Department of Biosciences, International Livestock Research Institute, Regional Office, 298 Kim Ma Street, Ba Dinh District, Hanoi, 100 000 Vietnam; 2 Department of Clinical Sciences, Swedish University of Agricultural Sciences, PO Box 7054, SE-750 07 Uppsala, Sweden; 3 Zoonosis Science Centre, Uppsala University, Po Box 582, SE-751 23 Uppsala, Sweden; 4 Department of Biosciences, International Livestock Research Institute, Po Box 30709, Nairobi, 00100 Kenya

**Keywords:** Research ethics, Scientific writing, Plagiarism, Citations, Reference managing

## Abstract

**Background:**

Referencing is an integral part of scientific writing and professional research conduct that requires appropriate acknowledgement of others’ work and avoidance of plagiarism. University students should understand and apply this as part of their academic development, but for this, it is essential that supervisors also display proper research integrity and support.

**Methods:**

This study used an online educative questionnaire to understand the knowledge and attitudes of students and supervisors at two institutes in Europe and Africa. The results were then used to create discussion around education of students and faculty in workshops and lectures.

**Results:**

Overall, 138 students and 14 supervisors participated: most were Swedish (89) and Kenyan (11). Overall, 98% had heard about plagiarism, and 35% believed it was common. Only 45% had heard about self-plagiarism, and when explained what it was, 44.5% considered it morally wrong. Europeans and North Americans had more knowledge than other nationalities. Most (85%) had received some training on referencing, but there was little consensus about principles, with more than 30% considering it acceptable to cite a reference in a paper they had not read. Discussing these results and the questions in workshops was helpful; it was also clear that there was no consensus among supervisors on what constituted correct behavior.

**Conclusions:**

This survey shows a need for greater consensus on appropriate referencing, and that there is need for more discussions and training on the topic for both students and faculty.

**Electronic supplementary material:**

The online version of this article (10.1186/s41073-018-0054-2) contains supplementary material, which is available to authorized users.

## Background

Science is a cumulative endeavor, building on previous work. A core principle of scientific writing is that all statements that are not common knowledge, or directly derived from the study that is the subject of the paper, should be referenced [1]. In practice, the process of referencing is not without pitfalls: chief among these are failure to reference, incorrect referencing, referencing for an ulterior purpose, and inappropriate use of self-referencing.

Failure to reference the words of other authors is considered plagiarism, which is a form of cheating [2]. Some students have likely been copying, cheating, and taking shortcuts for as long as there have been schools; moreover, plagiarism has been looked upon differently in different cultures and at different periods of human history [3, 4]. While plagiarism encompasses ideas as well as words, the internet and widespread use of word processors have made copying of phrases and sentences dramatically easier and plagiarism has become a more common cause of retraction of publications [5]. This type of copying is variously referred to as linguistic plagiarism, plagiarism of text, textual copying, textual reuse, or text-based plagiarism [6, 7]. In this paper, we will refer to this as plagiarism.

While traditions of verbal *viva voce* examinations may help discover cases where students have not actually written the work they are presenting, an important response from academia to text-based plagiarism has been the development and application of software for plagiarism detection [8–10]. However, increased use of software may also cause students to use other ways of cheating, such as increased use of paying others to write [10]. There is recognition that the problem is widespread and increasing and an agreement that students should be inculcated with ethical principles and legal implications related to cheating and plagiarism [8]. However, the views and definitions of plagiarism vary both among teachers and students, and even when there is an agreement of the principles of what plagiarism is, it is hard to agree on tolerance levels or appropriate responses [11, 12].

How students view cheating and plagiarism has many determinants. These likely include the culture, both inside and outside academia; the capacity, personality and training of students; and the benefits for successful plagiarism versus the likelihood of, and penalties for, detection. Students struggling more with writing, as well as students writing in non-native languages, may be more prone to use unpermitted aids in accomplishing their tasks, which may be a reason why non-native English speaking students often have been incriminated in plagiarism [3, 4, 11, 13]. Text-based plagiarism may also be more common in contexts where academic corruption is more common or more perceived to be common [6, 7]. Moreover, the field of correct referencing is not always a clear-cut matter of wrong and right, and also, academics may have different views on issues such as appropriate referencing, what is common knowledge, and self-plagiarism. Self-plagiarism is generally viewed as a lesser offense, especially when the views on plagiarism are lenient [14].

The aim of this study was to generate evidence to help supervisors build the capacity of their students. The overall goal of this project was to improve the scientific writing of students, through the specific objectives of this survey of improving the understanding of the attitudes towards plagiarism and knowledge of referencing.

## Methods

This study was conducted from September to October 2016, among students and supervisors predominately in agricultural sciences in order to assess the knowledge, attitudes, and practices (KAP) of students with regard to referencing and plagiarism, especially text-based plagiarism. Since KAP is likely to be dependent on both culture and education, efforts were made to include students from different backgrounds and this was reflected in the study design and tool developed. An online survey was developed using Netigate (www.netigate.net, Sweden) and piloted on academic staff, after which it was optimized. The survey did not use tools previously developed by other surveys since it was meant to be also a teaching tool: participants were asked a question, and after answering the question, they were given an explanation, in order to make it a learning opportunity. For example, the participants were asked if they had heard about plagiarism and if they knew what it was, and after answering this, they were given the definitions but had no possibility to go back and correct their answers. Definitions used were on purpose as simple and non-academic as possible, and thus Wikipedia definitions were used, as they were considered understandable by all participants. The study design and the questionnaire were approved by the International Livestock Research Institute (ILRI) Institutional Research Ethics Committee (ILRI-IREC 2015-14). Personal information is not published, and thus consent for publication is not applicable, but at the start of the survey, participants were informed of the purpose of the study and that they gave their consent to participating by clicking further (Additional file 1).

The survey used the following definitions, which was explained to the participants after the first questions regarding if they had heard about plagiarism and could describe it themselves:

**Table:** 

Plagiarism: Plagiarism is when you take the work or the text of someone else and pretend it is your own creation or writing. It is sometimes referred to as theft of intellectual material and is considered a form of cheating at universities. Wikipedia defines plagiarism as “Plagiarism is the ‘wrongful appropriation’ and ‘stealing and publication’ of another author’s ‘language, thoughts, ideas, or expressions’ and the representation of them as one’s own original work” The Swedish University of Agricultural Sciences defines it as “Plagiarism is when someone uses the work or text of another without clearly marking that it is someone else’s work.” https://www.slu.se/en/subweb/library/write-and-cite2/writing-references/cheating-and-plagiarism/ Self-plagiarism: Self-plagiarism is when you copy text that you have already written somewhere else. Wikipedia defines self-plagiarism as “the reuse of significant, identical, or nearly identical portions of one’s own work without acknowledging that one is doing so or without citing the original work”	

The online questionnaire (Additional file 1) was distributed through a mailed link to the following groups (only on one occasion), with the instruction that the survey was meant for students at master level and above and their supervisors:

1. Post-graduate students at the Swedish University of Agricultural Sciences (SLU) (approximately 500 students in the email lists).
2. Supervisors at the faculty of veterinary medicine and animal sciences at SLU (unknown number).
3. Undergraduate students at the veterinary program at SLU, during the final year (approximately 100 students in the email list).
4. Post-graduate students and supervisors working at the International Livestock Research Institute (ILRI), with main office in Kenya (approximately 100 persons in the email list).

The approach of collecting data through an online survey reduced the control over the number of people that the questionnaire reached, and no sample size calculation was done, since this approach did not allow control of the number of invited participants.

Results from the surveys were downloaded into MS Excel and analyzed descriptively. Nationalities and country of study were classed as either European/North American or other nationalities, and this was used to compare the attitudes towards plagiarism, using STATA 14 (STATACorp, USA) chi-square test, Fisher’s exact test, and *t* test when applicable. References were managed with Mendeley software (Mendeley Ltd).

After the results had been analyzed, the results were used as a tool to start discussion in workshops on scientific writing held by the author. A qualitative summary of the effectiveness of this is included in the results and the discussion.

## Results

Altogether, the email lists comprised around 1000 potential participants. The mailed survey was answered by 152 respondents (less than 20% participation rate), although 23 did not finish the full questionnaire; 138 (90.8%) reported to be students and 14 to be supervisors. The majority of the respondents were Swedish (89, 58.9%); this includes one person who reported dual nationality. The second most common nationality was Kenyan, with 11 respondents (7.3%); there were four Canadians and four Ethiopians. The remaining nationalities were Argentinian (2), Austrian (1), Bolivian (1), British (2), Burkinabe (1), Cameroonian (1), Chinese (1), Colombian (1), Costa Rican (1), Finnish (2), French (1), German (3), Ghanaian (1), Icelandic (1), Indian (2), Indonesian (1), Iraqi (1), Israeli (1), Italian (1), Mozambican (2), Nigerian (1), Norwegian (1), Polish (1), Rwandan (1), Slovakian (1), Spanish (3), Swazi (1), Ugandan (2), American (3), and Zimbabwean (1). All Swedish nationals stated that they either studied, or received their degree, in Sweden. For purposes of the comparisons, the nationalities were grouped into European/North Americans and others. Out of the 38 others, 20 were either studying or had obtained their most recent degree from a high-income country.

The academic experience was varying among the participants. Of the 14 supervisors, 11 had PhD degrees, 2 were still pursuing PhD degrees, and 1 was pursuing a master’s degree. Among students, 11 said that they were not pursuing degrees at the moment (4 had master’s degrees, 7 had bachelor’s degrees), 44 were pursuing master’s degrees, and 74 were pursuing PhD degrees. Apart from 21 of the students, all participants had written a thesis of some kind, of which 20 reported to have written a PhD thesis, 86 had written a master thesis and 79 a bachelor thesis. Seventy-six participants had never been an author of any peer-reviewed publication. Apart from one supervisor (who also had no PhD degree), all of those that had never written a peer-reviewed publication were students.

The respondents were asked if they had heard about plagiarism, and 140 out of 143 (98%) responded that they had, with significantly (*p* = 0.017) more European/North Americans (100%) than others (91.9%) (Table 1). When asked if they could briefly state what they thought it meant, all answers referred to copying of text or ideas without attributing it to the source.

**Table 1:** Awareness and perceptions about plagiarism among students and supervisors

	All participants	Female	Male	European/North American	Other nationalities
Participants	152	98	53	113	38
Age	31.3 (SD 7.6)	30.1 (SD 7.1)	33.8 (SD 8.1)	30.2 (SD 7.6)	34.5 (SD 7.0)
Ever authored or co-authored a peer-reviewed publication	76/152 (50%)	59/98 (60.2%)	16/53 (30.2%)***	66/113 (58.4%)	10 /38 (26.3%)***
Ever heard about plagiarism?	140/143 (97.9%)	89/91 (97.8%)	50/51 (98.0%)	105/105 (100%)	34/37 (91.9%)*
How common do you think it is?					
*I know many people doing this*	2/138 (1.5%)	1/88 (1.1%)	1/49 (2.0%)	1/102 (1.0%)	1/35 (2.9%)
*I think it is rather common*	48/138 (34.8%)	30/88 (34.1%)	18/49 (36.7%)	32/102 (31.4%)	15/35 (42.9%)
*I have heard it happens, but don’t think it is very common*	85/138 (61.6%)	54/88 (61.4%)	30/49 (61.2%)	67/102 (65.7%)	18/35 (51.4%)
*I have never heard about people doing this*	3/138 (2.2%)	3/88 (3.4%)	0/49 (0%)	2/102 (2.0%)	1/35 (2.9%)
What do you think is the most common reason to why people plagiarize?					
*Because of time constraints*	39/138 (28.3%)	27/88 (30.7%)	11/49 (22.5%)	33/102 (32.4%)	6/35 (17/1%)
*Because they feel they cannot write as well as others*	27/138 (19.6%)	14/88 (15.9%)	13/49 (26.5%)	15/102 (14.7%)	12/35 (34.3%)
*Because they do not know it is wrong*	26/138 (18.8%)	18/88 (20.5%)	8/49 (16.3%)	20/102 (19.6%)	6/35 (17.1%)
*Other reasons*	46/138 (33.3%)	29/88 (32.9%)	17/49 (34.7%)	34/102 (33.3%)	11/35 (31.4%)
Do you think this was a useful exercise?					
*Yes, it was somewhat useful*	68/129 (52.7%)	45/82 (54.9%)	23/47 (48.9%)	55/93 (59.1%)	13/35 (37.1%)
*Yes, I found it very useful*	38/129 (29.5%)	21/82 (25.6%)	17/47 (36.2%)	18/93 (19.4%)	20/35 (57.1%)***
*I wish I had done it before*	3/129 (2.3%)	3/82 (3.7%)	0/47 (0%)	3/93 (3.2%)	0/35 (0%)
*No, this is nothing new*	7/129 (5.4%)	5/82 (6.1)	2/47 (4.3%)	6/93 (6.5%)	0/35 (0%)
*Not useful for me, but I think others may benefit*	13/129 (10.1%)	8/82 (9.8%)	5/47 (10.6%)	11/93 (11.8%)	2/35 (5.7%)

*Significantly different (*p* < 0.05) compared to the comparison group to the left

***Significantly different (*p* < 0.001) compared to the comparison group to the left

In total, 89 out of 138 people responded that they had heard of someone plagiarizing; the most common answer as to how often it occurred was that they had heard of it but did not think it was very common. However, as many as 48 respondents stated that they thought it was rather common (Table 1). The most common single reason given for plagiarizing was time constraints, but ignorance was another commonly perceived reason, and also stated when respondents could give their own explanation. Other common reasons cited here were laziness, lack of morals, not caring, or similar.

When asked if there were instances when plagiarism could be acceptable, most replied that it was never acceptable, while some believed it could be acceptable in some cases when it was self-plagiarism. Some expressed that it was wrong but should in many cases not be punishable, and one respondent, a Swedish veterinary student, suggested that plagiarism occurs when supervisors have very high expectations of their students which drives the students into desperation; thus, you cannot fully blame the students for doing it.

**Fig. 1:**
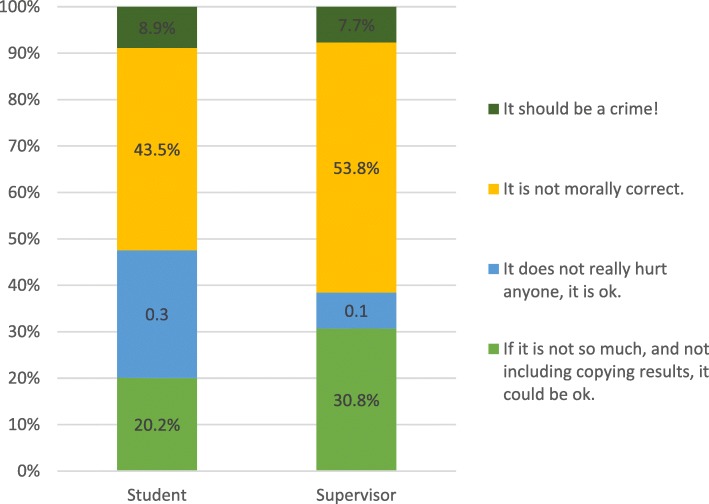
Attitudes towards self-plagiarism among students and supervisors

Out of 137 respondents, 62 (45%) had heard about self-plagiarism, and most answering the question considered it wrong; the most common answer was that it was morally wrong (Fig. 1). There was no difference between European/North Americans and others in this and no difference in terms of country of study.

**Fig. 2:**
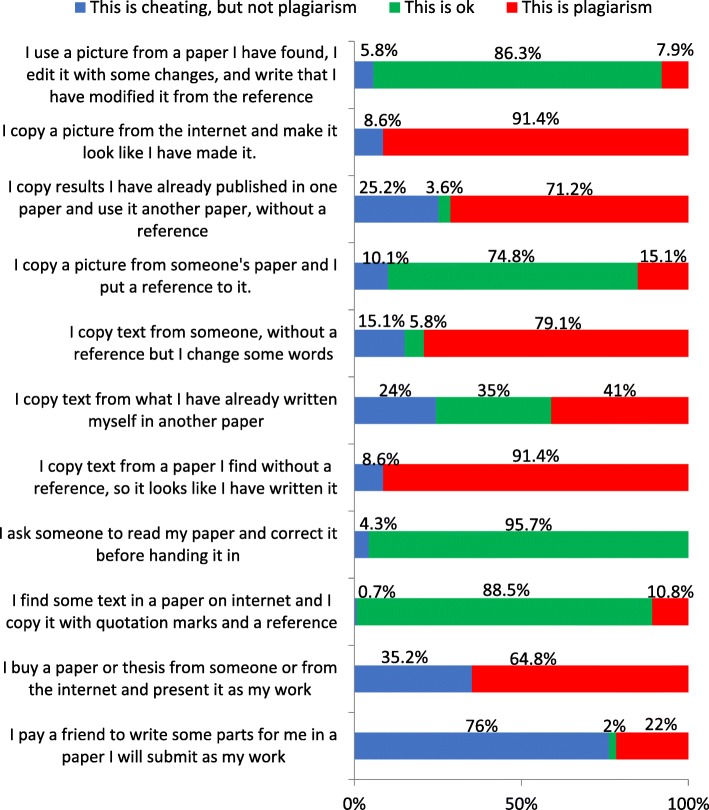
The attitudes of students and supervisors towards different scenarios

Attitudes towards different scenarios were judged by giving the participants a number of cases and asking them to judge whether each case constituted plagiarism, constituted cheating but not plagiarism, or was an acceptable practice (Fig. 2). There was a difference between European/North American nationals and others as to whether it was acceptable to pay someone to write part of a thesis (0.98% and 5.56% respectively), but this was not significant at *p* of 0.05 (*p* = 0.066). Significantly, more others (*p* = 0.01) considered it was plagiarism to copy text with quotation marks and reference. Significantly, more others (*p* = 0.037) considered it acceptable to copy text from someone, without a reference and changing some words (13.9% compared to 2.9% among Europeans/North Americans). For some additional questions, there were differences as to whether something was considered plagiarism or cheating but not as whether it was acceptable or not acceptable.

Most respondents were aware about means of detecting plagiarism: 93 said teachers could search the internet for suspicious text, and 128 knew that there was a software for detecting plagiarism. However, six respondents thought plagiarism can only be detected if you do it badly, and one student thought it could never be proven. Forty students did not know if there were any mechanisms for detecting plagiarism at their university, five said there were no mechanisms, 55 believed plagiarism was tested for all the time, and 35 believed it was tested for sometimes. There was a general consensus among all 135 answering the question that testing should be done, with 74% believing it should always be done; although this percentage was higher among students (Table 2).

**Table 2:** Perceptions about plagiarism and referencing among students and supervisors

	All participants	Students	Supervisors
Participants	152	98	53
Age	31.3 (SD 7.6)	30.2 (SD 6.6)	42.5 (SD 8.4)
In your opinion, should supervisors check for plagiarism?			
*Absolutely, always*	100/135 (74.1%)	94/122 (77.1%)	6/13 (46.2%)
*Maybe in suspected cases*	35/135 (25.9%)	28/122 (22.9%)	7/13 (53.8%)
What do you think is the most common reason to why people plagiarize?			
*Because of time constraints*	39/138 (28.3%)	38/125 (30.4%)	1/13 (7.7%)
*Because they feel they cannot write as well as others*	27/138 (19.6%)	24/125 (19.2%)	3/13 (23.1%)
*Because they do not know it is wrong*	26/138 (18.8%)	22/125 (17.6%)	4/13 (30.8%)
*Other reasons*	46/138 (33.3%)	41/125 (32.8%)	5/13 (38.5%)
Do you think this was a useful exercise?			
*Yes, it was somewhat useful*	68/129 (52.7%)	61/117 (52.1%)	7/12 (58.3%)
*Yes, I found it very useful*	38/129 (29.5%)	35/117 (29.9%)	3/12 (25.0%)
*I wish I had done it before*	3/129 (2.3%)	3/117 (2.6%)	0/12 (0%)
*No, this is nothing new*	7/129 (5.4%)	6/117 (5.1%)	1/12 (8.3%)
*Not useful for me, but I think others may benefit*	13/129 (10.1%)	12/117 (10.3%)	1/12 (8.3%)
What paper is generally the best one to refer to, in your opinion?			
*A literature review*	20/134 (14.9%)	18/121 (14.9%)	2/13 (15.4%)
*The oldest original study I can find*	51/134 (38.1%)	46/121 (38.0%)	5/13 (38.5)
*The latest paper in a topic*	63/134 (47.0%)	57/121 (47.1%)	6/13 (46.1%)
If you read B et al. 2006, you will find a reference to a book by B, 2004. B, 2004 refers to C et al.. 1964. C had actually done the research on this. How would you reference this?			
*The virus is economically important (A, 2012; B* et al*, 2006; B, 2004)*	121/134 (90.3%)	108/121 (89.3%)	13/13 (100%)
*The virus is economically important (C, 1964)*	13/134 (9.7%)	13/121 (10.7%)	0/13 (0%)

### Referencing

The majority, 123 out of 145 respondents, had received some training on referencing, but only 9 had taken a specific course, whereas the others reported it was part of a course, or a seminar. Fewer (76) reported to have used a referencing software, of which five did so without any training. The most commonly used referencing software was Endnote, with 69 users, followed Mendeley (21 users), and Zotero (12 users). The use of Mendeley was significantly (*p* < 0.001) higher among others.

When asked about why one should reference to peer-reviewed journals, the most common answers were that it was important to give credit where it was due, to find legitimate and best quality references, and to help the reader find the reference. There was no consensus about which paper was generally the best to use as a reference (Table 2), and almost as many people wanted to refer to the latest paper on a topic as to those who wanted to refer to the original research done.

All supervisors and most students considered a reference to multiple new papers more reliable than an old reference.

In addition, the participants were asked the following question:

“In a paper by A (2012) you can find the following statement: *‘The virus is considered an important economic pathogen in pigs (B et al 2006)’*. How can you reference this?”

Two students stated that it could be referenced to as it being well-known (one of them stated that it would be necessary to read the 2006 paper before making this statement) (Table 3). Other incorrect responses were that they would cite paper B without reading it (24 respondents), and they would cite both papers (22 respondents). All but one supervisor (male, from the UK) said that they wanted to read the original publication before citing, and one (male, from the USA) was willing to cite paper A as well as paper B.

**Table 3:** Answer from students and supervisors on the questions: “In a paper by A (2012) you can find the following statement: ‘The virus is considered an important economic pathogen in pigs (B et al 2006)’. How can you reference this?”

Statement	Participants	
The virus is economically important (A, 2012)	6	3.3%
The virus can be economically important (B et al. 2006)	40	22.3%
The virus is economically important (A, 2012; B et al. 2006)	22	12.3%
It is well known that the virus is economically important.	2	1.1%
I have to read B et al. 2006 before citing.	109	60.9%

Lastly, the participants were asked if they perceived this survey and if the content was a useful exercise, and the majority of both students and supervisors found it somewhat or very useful (Tables 1 and 2). There were significant differences between the categories of nationalities (*p* = 0.001). Only European/North Americans (7 in total) answered that the survey contained nothing new, and significantly more answered that the survey was not useful to them but maybe for others (12 European/North Americans answered this versus 1 other nationality).

### Training

As indicated in Tables 1 and 2, many participants in the surveys found it very or somewhat useful and the questions as well as the results have been used in three workshops in India as well as in lectures in Kenya. The format of asking questions first then providing definitions and answers, worked well in the workshop format with much time for discussions, but less well for a lecture format since it created much discussion and more time should have been put aside for this. However, the topic was popular both among students and supervisors, and the presentations were disseminated upon request.

## Discussion

In this survey, the attitudes and perceptions of students and supervisors about text-based plagiarism and referencing were explored. Although the study included a relatively small number of participants, it points to knowledge gaps and attitudes that could affect the quality of scientific writing. The survey used an educative approach whereby participants learned definitions and aspects of referencing as they progressed in the survey, which is different from other previous used validated tools for evaluating attitudes towards plagiarism, both in high-income and low- and middle-income countries [14–16].

Although small, the survey found a difference between European/North American and other nationalities in knowledge and attitudes towards plagiarism, which is in accordance with earlier studies [3, 4, 11–13]. However, this difference was not large and, for several issues, not significant, possibly due to the low power of the study. Nationals outside Europe and North America had less knowledge about plagiarism and did not consider some examples given the same way as European/North American nationals did. They did consider the exercise useful. The fact that 20 from the other nationality group had their training in high-income countries means that analyses by nationality is likely to bias the results towards null. The classification into two categories is admittedly a blunt tool, since large differences occur also within the European countries in regards to perceptions and handling of plagiarism issues, as shown by a previous survey on European institutes using an Academic Integrity Maturity Model [8]. In that survey, Sweden ranked third out of 27 surveyed countries and thus the high number of participants from Sweden means that these findings are not representative for all countries. The make-up of our respondents did not permit accurate comparison between European or other nationalities but still gives interesting insights into differences in attitudes.

Reasons given for plagiarism ranged from lack of time, laziness, or ignorance. Studies have shown that teachers often differentiate between intentional and unintentional plagiarism [12]. The fact that it is difficult for students to understand correct referencing implies some cases are unintentional. Where the rewards from plagiarism are high and the risks for detection and punishment are low, we would expect plagiarism to flourish. For example, in China, researchers were given monetary incentives for publishing in international journals; while this apparently increased overall publication rates, it is also believed to have fostered plagiarism (see [17] for details and references). In light of this, recent moves by some universities to require students to publish before they can obtain their MSc or PhD provides an incentive to publish at all costs including perhaps plagiarism or poor referencing.

In this context, the likelihood of detection and punishment for plagiarism are important disincentives. However, some teachers are concerned that detecting and punishing plagiarism may have negative consequences for them personally or for their university as a result of adverse publicity [12]. Other reasons not to pursue plagiarism cases officially include the administrative burdens of preparing the case, the high risk of students not being held accountable, and that teachers were often recommended not to pursue cases. It has also been argued that the whole concept of plagiarism as a punishable offense hinders a student-centered approach to teaching and that the concept should be abolished [9].

**Fig. 3:**
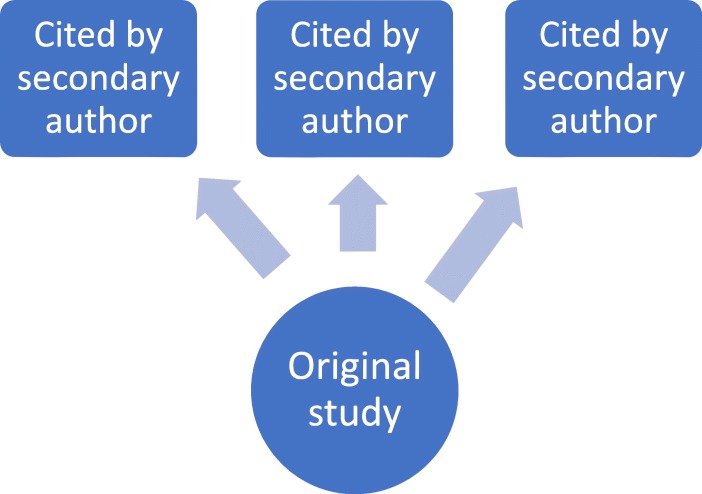
The case of one original study cited by multiple other papers

Lack of correct referencing skills has been noted earlier in connection with the issue of plagiarism [11]. Since plagiarism and correct referencing are to some degree interlinked, this study also included aspects of correct referencing as an indicator of respondents understanding and this revealed issues of misconceptions and disagreement both among student and supervisors. When an author is referencing citations by secondary authors, as illustrated in Fig. 3, there is a risk that the results of one study appear more solid when it has multiple references. As was clear in this study, all supervisors and most students would consider a statement more reliable if it contained multiple references. It was also evident that many were willing to cite a secondary reference, even without reading the original study, or to add both as references, which would give a false sense of reliability and risks citation out of context. There also seems a lack of understanding of the primary origin and purpose of referencing: supporting statements of fact and, hence, building a logical chain of evidence.

**Fig. 4:**
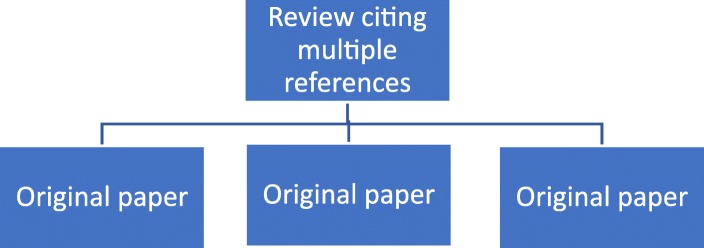
A review paper with multiple references

As illustrated in Fig. 4, one review contains multiple original references and a student may be tempted to cite the original papers without reading them, which may create an illusion of the student having read more papers than was the real case, in a way a fraudulent behavior [11]. Obviously, when papers are referenced without actually reading them, there can be no critical evaluation of the studies. Moreover, the context of the original study is not understood; for example, a study may report on a disease in a small number of sheep of a rare breed during a period of highly unusual climatic conditions, and this can be cited as a prevalence of disease in sheep generally.

Authors may be tempted to add multiple citations because of pressure on students to show that they have read more papers, but there may also be a deliberate wish to make a statement seem more reliable than it actually is, or a well-motivated desire to be comprehensive in citations. In addition, some journals or peer reviewers may promote the use of recent references instead of original, which may in fact discourage optimal referencing.

The majority of participants considered the exercise useful, perhaps indicating that issues of plagiarism and referencing are discussed too little among students and supervisors. Even though some indicated that they learned nothing new, the fact that there were different opinions about what to cite among supervisors as well as students indicate a need for more discussions also among senior scientists. For younger students, working with scenarios and examples may be a way of explaining the consequences of incorrect referencing.

Courses and trainings on research ethics or scientific writing should include aspects of referencing and plagiarism, since these are vital parts of academia. Correct referencing gives credit where it is due, helps the reader find further evidence, and gives weight behind statements allowing fact-based claims. Incorrect referencing can cause misperceptions and makes it difficult for the reader to know the truth behind statements, and therefore, it is an important ethical issue. Research ethics training was found to be more common in Pakistani students and faculty that had been trained abroad than within Pakistan but had no impact on attitudes towards plagiarism, whereas training in medical writing had an effect [14].

While this survey found some interesting differences in attitudes between different groups, there are several limitations. The definitions used in the online questionnaire included words such as “stealing” which may cause students to consider it more serious than they would have elsewise. In addition, the participants from different countries were not representative nor enough to allow detailed conclusions on cultural difference. The low-response rate, which could have been improved by sending out reminding emails, is a limitation of the study. Most participants in this survey, however, found it helpful, which may indicate that there is too little emphasis on the topics of plagiarism and referencing in the curriculum and also too little discussions in the academia.

### Piloting the use of these results as discussion tools

Using the questions asked in this survey on attitudes towards plagiarism and cheating was an efficient way of starting discussions, particularly when holding workshops including both supervisors and students. In one workshop, a supervisor maintained that he had no problem with students paying others to write their theses for them, since that was the way scientists published papers, which sparked a debate on the ethical aspects of having publications written by paid authors that are not on the author list. The questions on which paper to cite also served a good purpose to start the discussion about what to do when publications are behind pay walls, which can create problems, particularly for researchers in low-income countries [18].

## Conclusions

This small survey used an educative online survey to assess attitudes and perceptions towards plagiarism and referencing, with the aim of facilitating training in scientific writing. Training on these aspects may be particularly necessary in low-income countries and when English is not a native language, since the increased pressure of writing may push students into taking short cuts, even when they know it is wrong.

Surprisingly, there seems to be a lot of misperceptions as to how referencing should be done, even among supervisors. This indicates a need for more thorough training at different stages of the academic careers and more discussions within faculty. Research integrity may be difficult to teach students when supervisors do not always constitute good examples. The overall conclusion of this study is that these topics should be more frequently addressed and discussed and that the approach used for these questions can be used as a learning tool.

## Additional file

Additional file 1: Online questionnaire used in the survey. (PDF 107 kb)

## Abbreviations

ILRI: International Livestock Research Institute
SLU: Swedish University of Agricultural Sciences
