# Supplementary material for: Students’ and supervisors’ knowledge and attitudes regarding plagiarism and referencing
Source: Res Integr Peer Rev. 2018 Oct 23;3:10. doi: 10.1186/s41073-018-0054-2 (PMC6198419; doi:10.1186/s41073-018-0054-2)
Supplement: Supplementary file 1 — Online questionnaire used in the survey. (PDF 107 kb) [file 41073_2018_54_MOESM1_ESM.pdf]

Welcome to this survey on students' and supervisors' perceptions about plagiarism and referencing!

The purpose of this study is to collect information from students and supervisors about their knowledge and attitudes to plagiarism and referencing. The aim is to increase the knowledge in order to help supervisors and
teachers improve how they teach and discuss about these topics. At the same time, this survey will give you an opportunity to learn some about the topic, and improve your own skills while helping others.

Sometimes there is no answer that is completely right, and then we are more interested in your opinion on what is the best alternative.

Participation
in this study is voluntary and the results will be handled completely
anonymously. If you participate in the survey by clicking next, you approve that the results can be used for the purposes stated above.

If there are any questions about this survey, please don't hesitate to contact the responsible researcher:

Johanna Lindahl

Johanna.lindahl@slu.se

Thank you for your help!

1. First some demographic questions:

What is your nationality?

____

How old are you?

____

What gender are you?

- ☐ Female
- ☐ Male
- ☐ Other

In what country are you studying, or from which country have you gotten your last degree?

____

Are you a student or a supervisor?

- ☐ Student
- ☐ Supervisor

Which is your field of studies?

- ☐ Social science
- ☐ Natural science
- ☐ Mixed natural and social science
- ☐ Other ________________________

What is your topic of study? Example: Veterinary, Public health

____

What degree are your pursuing?

- ☐ Bachelor degree
- ☐ Master degree
- ☐ PhD
- ☐ Other
- ☐ I already have a PhD, am not pursuing a degree
- ☐ I already have a master, am not pursuing a degree

How many years university studies do you have (including the year you are in now)?

____

2. Now we would like to know more about your experiences in writing:

Have you ever written a thesis? Tick all that apply

- ☐ No
- ☐ Yes-bachelor thesis
- ☐ Yes- master thesis
- ☐ Yes- PhD thesis
- ☐ Yes-other ________________________________

Have you ever published a peer-reviewed paper? Tick all that apply

- ☐ No
- ☐ Yes- as first author
- ☐ Yes- as co-author

Have you ever published some other report or publication? Tick all that apply

- ☐ No
- ☐ Yes- as first author
- ☐ Yes- as co-author

3. We would like to know how much training you have in writing:

Have you ever had any training in referencing?

- ☐ No
- ☐ Yes, it was mentioned as part of a course
- ☐ Yes, some classes or seminars
- ☐ Yes, a course specific on this

Have you been trained on, or taught, how to use a reference handling system/ reference manager?

- ☐ No
- ☐ Yes

Which reference manager/managers have you learned?

____
____
____
____
____

4. Now we would like to discuss plagiarism:

Have you ever heard about plagiarism?

- ☐ Yes
- ☐ No

Could you describe in one sentence what it is?

____
____
____
____
____

5. We would like to know more about students attitudes to some issues:

Choose the alternative that you think is most applicable

	This is ok	This is plagiarism	This is cheating, but not plagiarism
I pay a friend to write some parts for me in a paper I will submit as my work	☐	☐	☐
I buy a paper or thesis from someone or from the internet and present it as my work	☐	☐	☐
I find some text in a paper on internet and I copy it with quotation marks and a reference	☐	☐	☐
I ask someone to read my paper and correct it before handing it in	☐	☐	☐
I copy text from a paper I find without a reference, so it looks like I have written it	☐	☐	☐
I copy text from what I have already written myself in another paper	☐	☐	☐
I copy text from someone, without a reference but I change some words	☐	☐	☐
I copy a picture from someone's paper and I put a reference to it.	☐	☐	☐
I copy results I have already published in one paper and use it another paper, without a reference	☐	☐	☐
I copy a picture from the internet and make it look like I have made it.	☐	☐	☐
I use a picture from a paper I have found, I edit it with some changes, and write that I have modified it from the reference.	☐	☐	☐

6.

Plagiarism is when you take the work or the text of someone else, and pretend it is your own creation or writing.

It is sometimes referred to as theft of intellectual material, and is considered a form of cheating at universities.

Wikipedia defines plagiarism as: "Plagiarism is the "wrongful appropriation" and "stealing and publication" of another author's "language, thoughts, ideas, or expressions" and the representation of them as one's own original work"

Swedish University of Agricultural Sciences defines it as "Plagiarism is when someone uses the work or text of another without clearly marking that it is someone else's work." http://www.slu.se/en/library/search/search-and-writers-guide/copyright-and-plagirism/what-are-cheating-and-plagiarism/

Have you heard about anyone doing this?

- ☐ Yes
- ☐ No

How common do you think it is?

- ☐ I know many people doing this
- ☐ I think it is rather common
- ☐ I have heard it happens, but don't think it is very common
- ☐ I have never heard about people doing this

What do you think is the most common reason to why people plagiarize?

- ☐ Because of time constraints
- ☐ Because they feel they can't write as well as others
- ☐ Because they don't think it is wrong
- ☐ Other reasons ___________________________________

In your opinion, are there any instances when plagiarism is ok?

____
____
____
____
____

7. Let's talk about self-plagiarism:

Have you heard about self-plagiarism?

- ☐ Yes
- ☐ No

Could you describe what it is?

____
____
____
____
____

8. Self-plagiarism is when you copy text that you have already written somewhere else.
Wikipedia defines self-plagiarism as "the reuse of significant, identical, or nearly identical portions of one's own work without acknowledging that one is doing so or without citing the original work"
How serious do you think this is? Choose the option that suits your opinion best.

	It should be a crime!	It is not morally correct.	It does not really hurt anyone, it is ok.	If it is not so much, and not including copying results, it could be ok.
Plagiarism	☐	☐	☐	☐
Self-plagiarism	☐	☐	☐	☐

9. Universities may take plagiarism more or less seriously, and in some cases there can be disciplinary actions. Journals also have rules about plagiarism.

How can a supervisor discover plagiarism? Tick all that apply
- ☐ They can google the text
- ☐ There are softwares to check this
- ☐ They can only detect it if you do it badly
- ☐ They can never prove it is plagiarized

Is this done by supervisors at your institution?
- ☐ No
- ☐ Yes, maybe sometimes
- ☐ Yes, it is a routine to do this all the time
- ☐ I don't know

In your opinion, should supervisors check for plagiarism?
- ☐ Absolutely, always
- ☐ Maybe good in suspected cases
- ☐ No

10. Now we have some questions about referencing:

Why should I try to reference peer-reviewed publications?

____
____
____
____
____

What paper is generally the best one to refer to, in your opinion?
- ☐ A literature review
- ☐ The oldest original study I can find
- ☐ The latest paper in a topic

11. Peer-reviewing is a system of letting other scientists critically evaluate the research of others to provide control of the standards of the work being published.
The purpose is to ensure that published research has used suitable methods for analyses, is relevant and merits publishing. It is not a guarantee that false data is not published since peer-reviewer seldom has access to raw data. It is also not a guarantee that the author references correctly.
Which of the following references would make you believe more in the statement?
- ☐ The virus is economically important (A, 2012; B et al, 2006; B, 2004)
- ☐ The virus is economically important (C, 1964)

12. There are some different opinions on how to reference

In a paper by A (2012) you can find the following statement "The virus is considered an important economic pathogen in pigs (B et al 2006). How can you reference this? Tick all that apply

- [ ] The virus is economically important (A, 2012)
- [ ] The virus can be economically important (B et al, 2006)
- [ ] The virus is economically important (A, 2012; B et al, 2006)
- [ ] It is well known that the virus is economically important.
- [ ] I have to read B et al 2006 before citing.

If you had read B et al, 2006, you would have found a reference to a book by B, 2004. B, 2004 refers to C et al. 1964. C had actually done the research on this. How would you reference this?

- [ ] It is correct to refer to either of A, 2012, B 2006, B, 2004, or C,1964
- [ ] I should include all references
- [ ] I should choose one of the later references, the first one is to old.
- [ ] I should refer to the oldest paper.
- [ ] I should read and refer to the paper that has original research.

13. When possible, always refer to original research and never refer to a paper you have not read. If you can only find a review, it is better to indicate it.

You can indicate that you have read a review by writing something like:

As reviewed by x (2014), most studies indicates that this is economically important.

By adding multiple references, all referring to the same original research, it may seem like more people have found the same results, making it more plausible.

If you cite a paper you have not read, you risk spreading the wrong information, because there is a risk that the paper you read misinterpreted the results.

14. In this survey we have covered plagiarism and referencing.

Do you think this was a useful exercise?

- [ ] Yes, it was somewhat useful
- [ ] Yes, I found it very useful
- [ ] I wish I had done this earlier
- [ ] No, this is nothing new
- [ ] No, I find this topic meaningless
- [ ] Not useful for me, but I think others may benefit

If you thought it useful, please forward the link to others that may want to answer it.

More examples of what is plagiarism or not can be found on the internet.

You can try to study the examples given by the Norwegian University of Life Sciences:

http://www.umb.no/statisk/noragric/writing_centre/2012/what_is_plagiarism.pdf

Thank you for participating, and we hope you learned something from it!

Please forward the link to friends and colleagues that you think might benefit from it.
